# Relationship Between Emotional Intelligence and Burnout Among Anesthesia and resuscitation Care Staff

**DOI:** 10.1192/j.eurpsy.2025.1575

**Published:** 2025-08-26

**Authors:** F. Chelly, I. Kacem, A. Fki, S. Chaima, N. Belhadj Chabbah, O. Ajmi, N. Ben Gadha, J. Ben Khelifa, S. Ksibi, H. Ben Hamada, M. Kahloul, W. Naija

**Affiliations:** 1 Faculty of Medicine Ibn Al Jazzar, University of Sousse; 2 Department of Occupational Medicine, Sahloul University Hospital; 3 Farhat Hached University Hospital, Department of Occupational Medicine; 4 Psychiatry Department, Farhat Hached University Hospital; 5 Anesthesia and Intensive Care Department, Sahloul University Hospital, Sousse, Tunisia

## Abstract

**Introduction:**

Anesthesia and critical care professionals are particularly vulnerable to burnout due to the demanding nature of their work, including long hours, night shifts, high-stress situations, and the constant risk of medical errors. Emotional intelligence (EI) has emerged as a crucial factor in fostering positive professional relationships and mitigating burnout.

**Objectives:**

To study the association between EI and burnout among healthcare workers in anesthesia and critical care settings.

**Methods:**

This is a cross-sectional descriptive and analytical study conducted over a period of 3 months in 2024. It included 72 healthcare workers in the anesthesia and critical care departments at the Sahloul University Hospital in Sousse. The survey was based on a self-administered questionnaire, including data on socio-demographic and professional characteristics, the Maslach Burnout Inventory (MBI), and the Schutte Self-Report Emotional Intelligence Test (SSEIT).

**Results:**

The majority of participants were female (58.3%) with an average age of 33 years. Most participants (61.1%) were nurses, and 40.3% worked in the surgical critical care unit. The average EI score was 103.26. Among the EI factors, emotion perception was the most affected, with a score of 30.69 ± 9.34. EI levels were low in 11.1% of cases. Severe burnout was present in 19.4% of participants, and 65.3% had a high emotional exhaustion (EE) score. Additionally, 70.8% had a high depersonalization score, and 58.3% exhibited low personal accomplishment. EI levels was statistically associated with time spent with family and friends (p = 0.027). Among the professional factors, job satisfaction was significantly associated with EI level (p = 0.001). The factors significantly associated with severe burnout were the absence of alcohol consumption (p = 0.000) and the absence of time spent with loved ones (p = 0.033). A significant relationship between EI and self-emotion management and EE was reported (p = 0.040).

**Conclusions:**

EI is crucial for preventing burnout among healthcare workers in anesthesia and critical care. By enhancing positive attitudes, managing stress, and improving interpersonal skills, EI can improve work life and patient care. Incorporating EI training into health science curricula can equip future providers with essential tools for well-being.

**Disclosure of Interest:**

None Declared

